# Vascular Access Surgery Timing: A Prospective European Cohort Study in Elderly Patients with ESKD

**DOI:** 10.34067/KID.0000001180

**Published:** 2026-03-27

**Authors:** Boudewijn D.C. Heggen, Nicholas C. Chesnaye, Friedo W. Dekker, Merel van Diepen, Joris I. Rotmans, Fergus J. Caskey, Claudia Torino, Antonio Vilasi, Maciej Szymczak, Christiane Drechsler, Christoph Wanner, Maria Pippias, Kitty J. Jager, Marie Evans, Mickaël Hiligsmann, Geert Willem H. Schurink, Maarten G.J. Snoeijs, The EQUAL Study Investigators, Andreas Schneider

**Affiliations:** 1 Department of Vascular Surgery, Maastricht University Medical Center+, Maastricht, The Netherlands; 2 ERA Registry, Department of Medical Informatics, Amsterdam UMC, University of Amsterdam, Amsterdam, The Netherlands; 3 Amsterdam Public Health Research Institute, Quality of Care, Amsterdam, The Netherlands; 4 Department of Clinical Epidemiology, Leiden University Medical Center, Leiden, The Netherlands; 5 Department of Internal Medicine, Leiden University Medical Center, Leiden, The Netherlands; 6 Population Health Sciences, Bristol Medical School, University of Bristol, Bristol, United Kingdom; 7 IFC-CNR, Clinical Epidemiology and Pathophysiology of Renal Diseases and Hypertension, Reggio Calabria, Italy; 8 Clinical Department of Nephrology, Transplantation Medicine and Internal Diseases, Wroclaw Medical University, Wroclaw, Poland; 9 Division of Nephrology, Department of Medicine, University of Würzburg, Würzburg, Germany; 10 Interdisciplinary Center for Palliative Medicine, University Hospital Würzburg, Würzburg, Germany; 11 Department of Clinical Research and Epidemiology, Comprehensive Heart Failure Center, University Hospital Würzburg, Würzburg, Germany; 12 North Bristol NHS Trust, Renal Unit, Bristol, United Kingdom; 13 Renal Unit, Department of Clinical Intervention and technology (CLINTEC), Karolinska Institutet and Karolinska University Hospital, Stockholm, Sweden; 14 Department of Health Services Research, CAPHRI Care and Public Health Research Institute, Maastricht University, Maastricht, The Netherlands

**Keywords:** arteriovenous access, clinical trial, dialysis access, hemodialysis, hemodialysis access, vascular access

## Abstract

**Key Points:**

- Only 32% of elderly patients with ESKD in Europe started hemodialysis with a functional fistula that was created on time.
- Vascular access was created early in 42% and late in 26% of patients, exposing these patients to unused fistulas or catheter dependency.
- Eventually, 90% of all patients after fistula creation started hemodialysis within the mean follow-up period of 30 months.

**Background:**

Timing vascular access creation for older patients with ESKD is challenging. Autologous arteriovenous fistulas (AVF) are often created too early, leading to unused fistulas, or too late, necessitating central venous catheters (CVC). This study reports on vascular access creation in relation to hemodialysis initiation in European practice.

**Methods:**

This study is a secondary analysis of the European Quality cohort: a prospective observational study that recruited patients aged ≥65 years with stage 4 or 5 CKD from six European countries. Patients were selected once their eGFR dropped below 15 ml/min per 1.73 m^2^, if they received a vascular access, and had at least 6 months of follow-up. Vascular access timing was categorized into three groups: early (no dialysis within 6 months after AVF creation), on time (dialysis initiated with an AVF within 6 months), and late (hemodialysis initiated with a CVC before or within 6 months after AVF creation).

**Results:**

A total of 332 patients from the European Quality cohort were analyzed: 221 patients (67%) received an AVF as first vascular access and 111 patients (33%) a CVC. Among the latter, 53 patients (48%) underwent subsequent AVF creation, whereas for the remaining 58 patients, CVC was considered the intended vascular access strategy. At dialysis initiation, 158 patients (54%) used an AVF, while 136 patients (46%) used a CVC. AVF creation was on time in 88 of 274 patients (32%), early in 114 patients (42%), and late in 72 patients (26%). Despite high rates of early AVF creation, most fistulas were eventually used, as 90% of patients initiated hemodialysis within 3 years.

**Conclusions:**

Only 32% of elderly patients with ESKD started hemodialysis with a functional AVF created on time (within the 6-month window), while 42% had early and 26% late creation. Clinical prediction tools are needed to optimize vascular access timing.

## Visual Abstract

**Figure:**
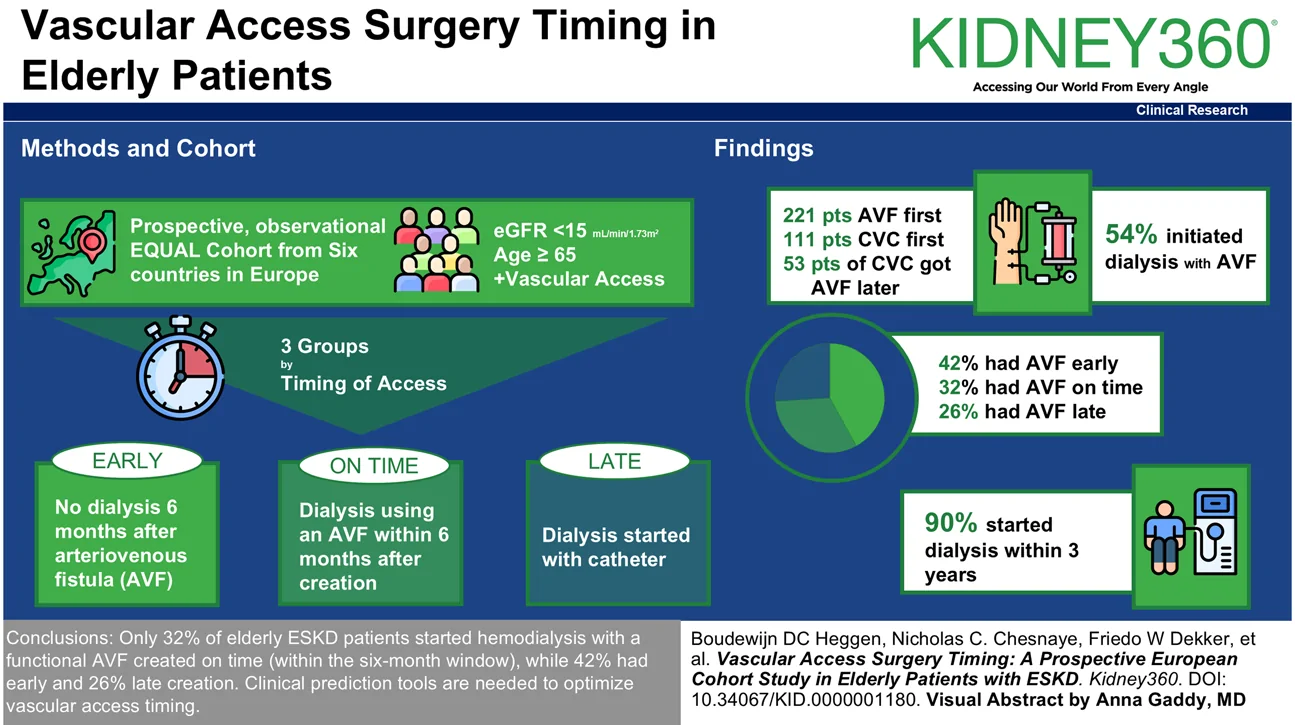

## Introduction

In Europe, over half a million patients with ESKD receive KRT, with a median age of 68 years for incident patients. For 83% of these patients, hemodialysis is the initial treatment modality.^1^ To facilitate hemodialysis treatment, patients need a functional vascular access, with an autologous arteriovenous fistula (AVF) being the preferred option.^2,3^ To allow time for maturation, guidelines recommend creating an AVF 3 to 6 months before the anticipated start of hemodialysis.^2^

Timing of AVF creation is challenging due to the unpredictable decline in kidney function and the variable onset of uremic symptoms or hypervolemia. Estimating the need for hemodialysis initiation 3 to 6 months in advance may therefore not always be feasible. As a result, vascular access planning involves a balance between creating the access early enough to ensure readiness while avoiding unnecessary procedures.

Creating an AVF well before hemodialysis initiation provides time for maturation and potential interventions to improve maturation, thereby increasing the likelihood of starting hemodialysis with a functional AVF. However, early AVF creation may also lead to unused fistulas as some patients die before hemodialysis initiation, while others receive a kidney transplant or change their mind and opt for conservative treatment. Furthermore, early AVF creation prolongs the exposure to possible vascular access complications such as hand ischemia.

When an AVF is created too late, patients may need a central venous catheter (CVC) to start hemodialysis treatment. This risk is more pronounced in elderly patients who face a 40% higher chance of nonmaturation and early failure.^4^ Although CVCs are helpful when hemodialysis has to start urgently, they are associated with increased mortality and bloodstream infection.^5^

There are limited data regarding the actual use of preemptive AVFs in elderly patients in European countries. A Canadian study found that 81% of adults with a preemptive AVF initiated hemodialysis during follow-up, with a median of 6.1 months. Nearly 30% started hemodialysis using a CVC. Older age was associated with a lower likelihood of starting hemodialysis.^6^ Similarly, a US study involving patients aged 70 years or older reported that 80% of predialysis vascular accesses were AVFs. In this cohort, 33% did not start hemodialysis within 2 years. Among those with preemptive AVF creation, 46% initiated hemodialysis with a CVC, and 33% of CVC users had a maturing AVF.^7^

With the aging population and rising numbers of elderly patients starting dialysis, understanding optimal timing of AVF creation has become increasingly urgent. Therefore, a large multinational cohort of elderly patients with ESKD was used to analyze current clinical practice in Europe. The objective of this study was to report on the timing of vascular access creation, with patients categorized as receiving vascular access creation on time (AVF creation within 6 months before hemodialysis initiation), early (hemodialysis not initiated within 6 months after AVF creation), or late (initiation of hemodialysis with a CVC within 6 months after AVF creation or before AVF creation). We compared patient characteristics across these groups and examined the long-term fate of AVFs created before dialysis initiation.

## Methods

### Study Design

We performed a secondary analysis of the European Quality (EQUAL) cohort data. EQUAL is a prospective, observational cohort study that recruits patients aged 65 years and older with an eGFR of 20 ml/min per 1.73 m^2^ or lower.^8,9^ The primary aim of EQUAL was to investigate the associations between level and trajectory of kidney function, uremic signs and symptoms, quality of life, patient preferences/decision making, and the timing of initiation of dialysis or other clinical outcomes over up to 4 years of follow-up. The EQUAL database includes information on the initiation of hemodialysis, vascular access procedures, and comorbidities. Ethical approval for the EQUAL study was obtained from the medical ethical committees or corresponding Institutional Review Boards of all participating centers.^9^ As this secondary analysis uses anonymized data from the EQUAL study, no additional ethical approval was required. For our study, we selected patients when their eGFR first fell below 15 ml/min per 1.73 m^2^, as this is the moment at which patients are generally referred for vascular access planning.

### Setting

Patients were recruited from nephrology clinics across six European countries (The Netherlands, Germany, Italy, Sweden, United Kingdom, and Poland) between March 2013 and August 2023.

Patients who met the eligibility criteria were consecutively approached. Follow-up assessments were conducted using data routinely collected during standard clinical care, with biannual assessments for patients in the predialysis phase and quarterly for those who had commenced dialysis.^8,9^ After 6 months of dialysis, the follow-up frequency reverted to 6-month intervals. Follow-up continued until the occurrence of one of the following: death, transition to primary care, or completion of 4 years of follow-up, whichever occurred first.^9^ Patients were considered lost to follow-up when they transferred to a different nephrology clinic or when they missed a follow-up assessment. Follow-up was also discontinued when a patient underwent a kidney transplantation.

### Participants

To create the Vascular Access Surgery Timing (VAST) cohort, we selected patients whose eGFR declined below 15 ml/min per 1.73 m^2^ and who underwent vascular access surgery during follow-up. Patients with missing information on the date of surgery or vascular access type were not considered for analysis. In addition, patients whose first access was an arteriovenous graft (AVG), or who opted for peritoneal dialysis, were not included in the cohort. Furthermore, patients were not selected for the VAST cohort if their follow-up ended within 6 months after the creation of an unused AVF, as vascular access outcomes could not be determined in these patients. Unless follow-up ended due to death or transition to another KRT, as these were considered relevant study end points. Similarly, patients whose follow-up ended within 6 months after CVC placement without subsequent AVF creation were excluded, unless follow-up ended due to death or kidney transplantation.

We divided the VAST cohort into clinically relevant groups based on the time from vascular access creation until hemodialysis initiation. Considering the European Society for Vascular Surgery guidelines, the upper limit of the recommended window (6 months) was used as a threshold to define three mutually exclusive groups^2^.

- Vascular access created early: no initiation of hemodialysis within 6 months following AVF creation (either due to persistent predialysis status or patient death).
- Vascular access created on time: initiation of hemodialysis using an AVF within 6 months after AVF creation.
- Vascular access created late: initiation of hemodialysis using a CVC within 6 months after AVF creation or hemodialysis initiation with a CVC before AVF creation.

In addition, there was a subset of patients who initiated hemodialysis with a CVC but did not receive an AVF during a follow-up period of at least 6 months. For these patients, it was assumed that the CVC was their intended vascular access.

### Data Sources and Variables

At inclusion and at the designated follow-up intervals, the EQUAL study prospectively gathered data. This encompassed information extracted from the patients' medical records and from physical examinations. Baseline characteristics, including age, gender, primary kidney disease, and comorbidities, were recorded at inclusion. At inclusion and follow-up assessments, information was recorded on BP, body weight, and height. Finally, data on blood and urine biochemistry, medication, and treatment characteristics as well as hospitalization and mortality were collected.

### Statistical Methods

Data were processed using IBM SPSS (version 28.0). Data are presented as mean±SD for continuous variables and as percentages for categorical variables. A chi-square test was used to determine if there were significant differences between the three groups for categorical variables. An ANOVA was used for noncategorical variables. The timeline after AVF creation was assessed using a cumulative incidence competing risk analysis, with initiation of hemodialysis and death as events.^10^ Censoring occurred at the time of the last follow-up or at 3 years, whichever came first, if the event of interest had not occurred.

Variables were manually inspected for outliers, missing values, and inconsistencies. Missing baseline characteristics and comorbidities were assumed to be missing at random and were therefore not imputed, as missing data for these variables accounted for <10%. Biochemical data were taken from the measurement at inclusion, except for eGFR, which was measured at the time of vascular access creation, or, if unavailable, up to 3 months prior. Missing biochemical data were also assumed to be missing at random and were not imputed. Missing data on hemodialysis initiation, vascular access creation, and operation dates were supplemented where possible using other available information, such as hospital admission dates, reasons for admission, and laboratory data. If the date of first hemodialysis was unavailable, it was assumed that patients with a CVC commenced hemodialysis on the day of catheter placement. For patients with an AVF and unknown hemodialysis start dates, the start date was imputed as the midpoint between the last predialysis measurement and the first dialysis measurement. If a patient received multiple AVFs, it was assumed that hemodialysis was performed using the most recently created AVF, as data on actual AVF use were not available. In cases where a patient received a CVC after AVF creation, it was assumed that hemodialysis was initiated using the catheter from the date of its placement.

## Results

### Participants

The EQUAL cohort comprised a total of 1736 subjects, of whom 1245 experienced a decline in eGFR to below 15 ml/min per 1.73 m^2^. Among these participants, 154 opted for peritoneal dialysis. In total, 521 patients received a vascular access for hemodialysis during follow-up. For 127 patients, the date of vascular access creation was unknown and for six patients an AVG was the first vascular access. In addition, 29 and 27 patients were excluded because they were lost to follow-up within 6 months after the creation of an AVF or CVC, respectively. Overall, 332 patients were selected in the VAST cohort (Figure 1).

**Figure 1:**
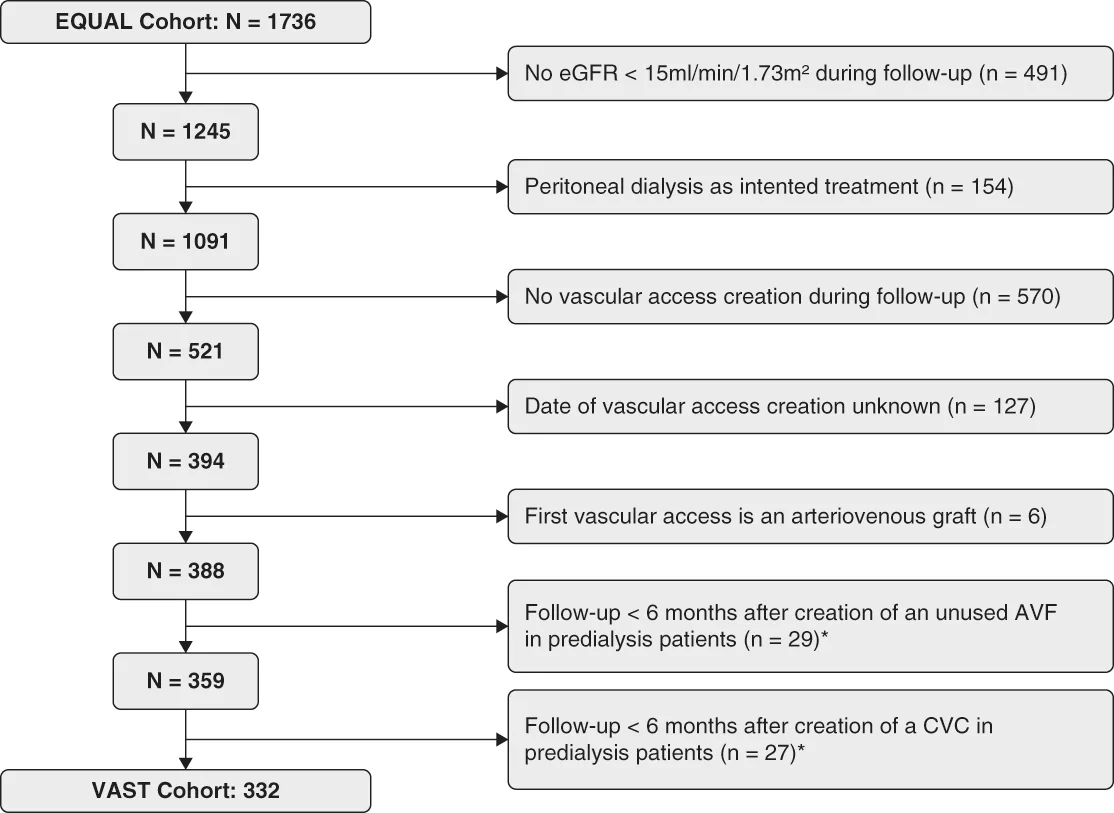
**Patient selection flow chart.** *Except if the follow-up ended because of death, switch to conservative therapy, or kidney transplantation. AVF, autologous arteriovenous fistula; CVC, central venous catheter; EQUAL, European Quality; VAST, Vascular Access Surgery Timing.

### Baseline Characteristics

Participants had a mean age of 76 years and a mean body mass index of 28.3 kg/m^2^, men comprised 77% of the cohort. Hypertension (28%) was the most common cause of ESKD, followed by diabetes mellitus (24%), glomerular disease (11%), and tubulointerstitial disease (9%). The mean follow-up duration was 29.5 months, with a total follow-up time of 815 patient-years (Table 1). During follow-up, 11 patients (3.3%) underwent kidney transplantation, after which follow-up was discontinued.

**Table 1:** Demographic data from the study cohort and subgroups based on timing of vascular access creation and central venous catheter strategy

Baseline	Total	AVF	AVF Created	AVF	CVC Intended
	*N*=332	Created Early	On Time	Created Late	Strategy
		*n*=114	*n*=88	*n*=72	*n*=58
Age (yr)	76±6.4	75±6	75±6	76±6	78±7
Sex (male)	77% (254)	76% (87)	84% (74)	74% (52)	70% (41)
BMI (kg/m^2^)	28.3±5.3	29.3±5.1	28.1±5.6	27.6±5.2	27.6±5.2
**Primary renal disease**					
Hypertension	28% (90)	32% (36)	22% (19)	22% (15)	35% (20)
Diabetes	24% (79)	24% (27)	19% (16)	26% (18)	31% (18)
Glomerular disease	11% (37)	6% (7)	15% (13)	19% (13)	7% (4)
Tubulo-interstitial disease	9% (30)	11% (12)	14% (12)	6% (4)	3% (2)
Miscellaneous	28% (91)	28% (32)	30% (26)	28% (19)	24% (14)
Predialysis mo to first AVF	−	−8.6±7.7	−9.5±9.2	−8.6±9.6	—
Mean follow-up (mo)	29.5±16.2	34.3±16.0	27.7±15.0	31.0±15.7	22.7±16.5
**Comorbidities**					
Hypertension	93% (297)	94% (104)	94% (79)	91% (62)	93% (52)
Diabetes	46% (150)	46% (52)	43% (37)	44% (30)	53% (31)
Current smoker	11% (36)	9% (10)	11% (10)	13% (9)	26% (15)
Myocardial infarction	17% (56)	15% (17)	14% (12)	17% (12)	26% (15)
Congestive heart failure	17% (54)	10% (11)	13% (11)	18% (12)	36% (20)
Peripheral vascular disease	16% (51)	13% (15)	13% (11)	12% (8)	29% (17)
Lung disease	13% (41)	10% (11)	9% (8)	16% (11)	19% (11)
Cerebrovascular disease	15% (50)	16% (18)	14% (12)	13% (9)	19% (11)
Malignancy^a^	22% (71)	22% (24)	33% (27)	19% (13)	12% (7)
**Biochemical**					
Albumin (g/dl)	3.7± 0.6	3.8±0.6	3.7±0.5	3.5±0.6	3.6±0.6
Serum bicarbonate (mEq/L)	22±4	22±4	21±3	22±4	24±4
Serum calcium (mg/dl)	9.0±0.8	9.2±0.7	8.9±0.6	8.9±0.9	8.9±0.9
Hemoglobin (g/dl)	11.2±1.5	11.7±1.6	11.1±1.3	10.9±1.4	10.7±1.5
Serum potassium (mEq/L)	4.7±0.6	4.7±0.6	4.8±0.6	4.6±0.7	4.5±0.7
Serum phosphate (mg/dl)	4.6±1.1	4.3±0.9	4.6±0.9	4.8±1.1	4.8±1.6
eGFR (ml/min per 1.73 m^2^)^b^	10±3	11±3	10±3	9±3	9±3
**Countries**					
Germany	38	21% (8)	34% (13)	32% (12)	13% (5)
Italy	89	27% (24)	29% (26)	17% (15)	27% (24)
The Netherlands	49	43% (21)	20% (10)	22% (11)	14% (7)
Poland	30	23% (7)	43% (13)	17% (5)	17% (5)
Sweden	50	36% (18)	20% (10)	28% (14)	16% (8)
United Kingdom	76	47% (36)	21% (16)	20% (15)	12% (9)

Demographics from the total study cohort, the three clinically relevant groups based on the time from vascular access creation until hemodialysis initiation, and the subset of patients for who a central venous catheter was considered the intended strategy. Vascular access created early: no initiation of hemodialysis within 6 months following vascular access creation. Vascular access created on time: initiation of hemodialysis with an autologous arteriovenous fistulas within 6 months following arteriovenous fistula creation. Vascular access created late: initiation of hemodialysis with a central venous catheter within 6 months after vascular access creation or hemodialysis initiation with a central venous catheter before autologous arteriovenous fistulas creation. Continuous variables are presented as mean±SD. Categorical variables are presented as percentages with absolute numbers. Reported *P* values for continuous variables are derived from an omnibus one-way ANOVA, and for categorical variables a chi-square test was used. *P* values were calculated to assess differences between the three autologous arteriovenous fistulas groups, without a reference group. The total cohort and the central venous catheter group are provided for descriptive purposes only and were not part of the statistical comparison. AVF, autologous arteriovenous fistula; BMI, body mass index; CVC, central venous catheter.

a Except basal cell and squamous cell carcinoma of the skin.

b eGFR was measured at the time of the first vascular access creation, or, if unavailable, up to 3 months prior.

### Timing of Vascular Access Creation

The first vascular access was an AVF for 221 patients (67%) and a CVC for 111 patients (33%). For 58 patients (52%), the CVC was considered the intended vascular access strategy, and for 53 patients (48%), an AVF was created after starting hemodialysis with a CVC. At hemodialysis initiation, 158 patients (54%) used an AVF and 136 patients (46%) used a CVC. Based on the predefined groups, vascular access was created on time in 88 of the 274 patients (32%) who received an AVF during follow-up. The AVF was created early in 114 patients (42%), while in 72 patients (26%) it was created late (Figure 2).

**Figure 2:**
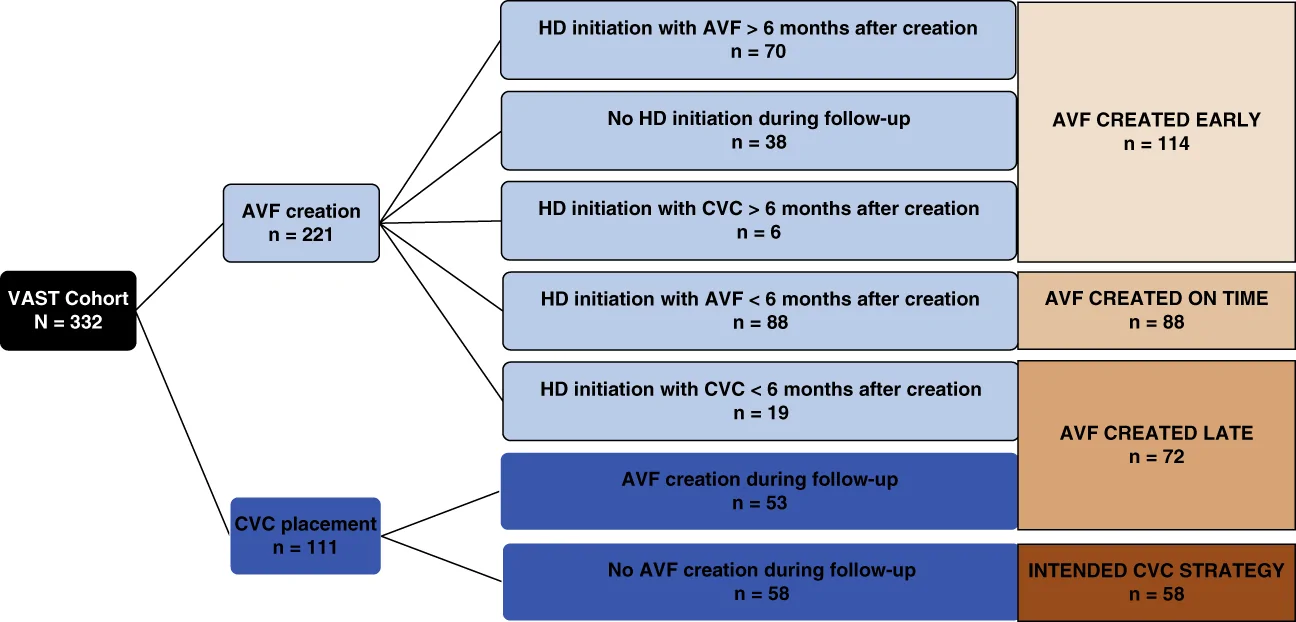
**Distribution of selected patients after the first vascular access was created.** HD, hemodialysis.

### Comparison of Patients with Different Timing of Vascular Access Creation

The mean age was similar across the three groups, with a slightly higher proportion of men in the on-time group (84%) compared with the early (76%) and late (74%) groups, although this was not statistically significant (*P* = 0.264).

Comorbidities were prevalent in all groups, with hypertension affecting 93% of patients and diabetes 46%, without significant differences across the groups (*P* = 0.75 and *P* = 0.93, respectively). The prevalence of cardiovascular diseases, including myocardial infarction (17%), congestive heart failure (17%), and peripheral vascular disease (16%), was similar across groups (all *P* > 0.05). Malignancy was more frequent in the on-time AVF group (33%) compared with early (22%) and late (19%) groups, but this did not reach statistical significance (*P* = 0.09).

Patients in the group where the AVF was created late had lower mean serum albumin levels (3.5±0.6 g/dl) compared with the early (3.8±0.6 g/dl) and on-time (3.7±0.5 g/dl) groups (*P* = 0.002). Similarly, serum calcium was highest in the early AVF group (9.2±0.7 mg/dl) and lowest in the late AVF group (8.9±0.9 mg/dl, *P* = 0.006). Hemoglobin concentrations were lower in the late AVF group (10.9±1.4 g/dl) compared with the early group (11.7±1.6 g/dl, *P* = 0.001). Serum phosphate levels were higher in the late group (4.8±1.1 mg/dl) compared with the early group (4.3±0.9 mg/dl, *P* = 0.001). Patients in whom the AVF was created late had a lower eGFR compared with those who underwent early AVF creation (9±3 versus 11±3 ml/min per 1.73 m^2^, *P* = 0.002).

There was no significant difference in vascular access timing between the six participating countries with the proportion of AVFs created early ranging from 21% to 47%, AVFs created on time ranging from 20% to 43%, and AVFs created late ranging from 17% to 32% (Table 1).

### Long-Term Outcome of Fistulas Created before Dialysis Initiation

Of the 221 patients who received an preemptive AVF, 114 patients belong to the AVF created early group, 87 patients to the AVF created on time group and 20 patients to the AVF created late group. Within 6 months 108 patients (48%) had initiated hemodialysis. One year after AVF creation, 148 patients (69%; 215 at risk) had commenced hemodialysis treatment, and 182 patients (90%; 203 at risk) had started hemodialysis after 3 years. Some of these patients used a vascular access different from the initial AVF, such as a newly created AVF or a CVC. Only 21 patients (10%) had not initiated hemodialysis treatment 3 years after AVF creation: 16 of these patients had died and the remaining five were alive without dialysis (Figure 3).

**Figure 3:**
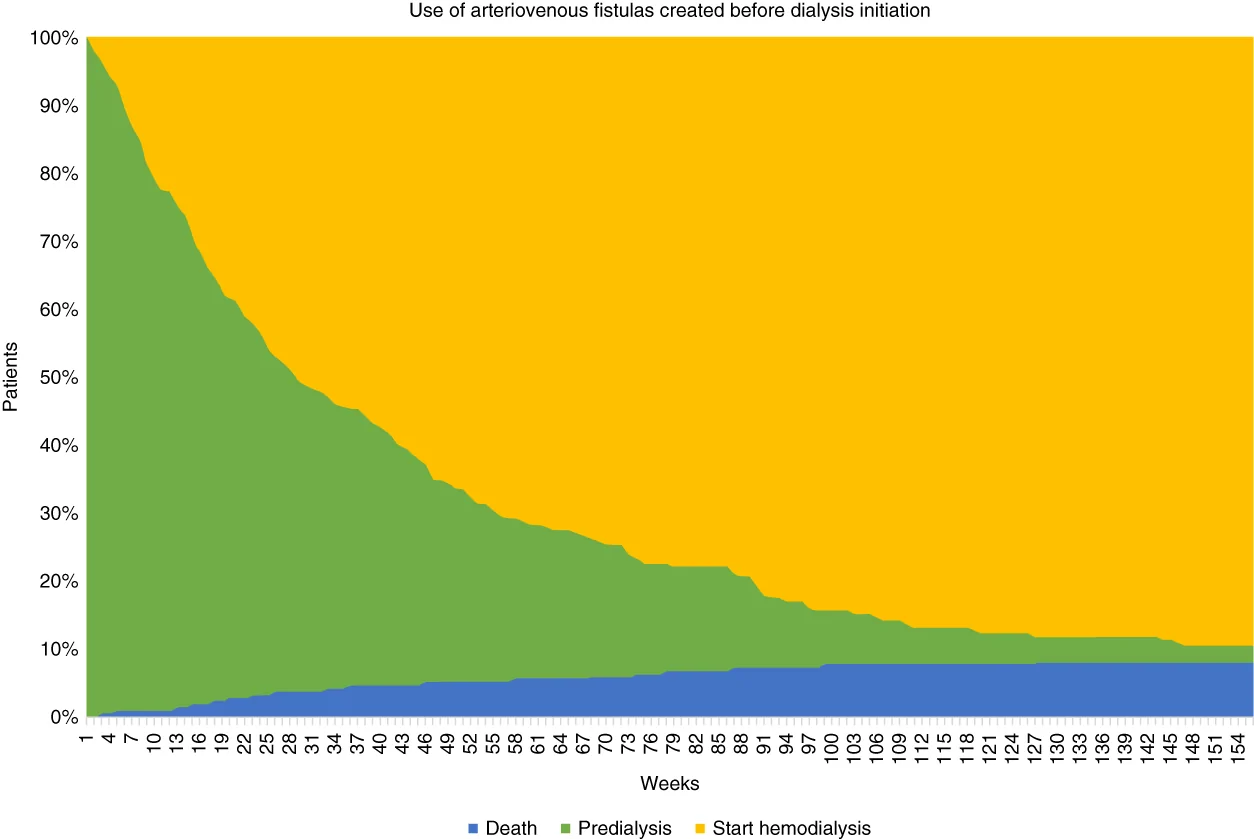
**Survival curve after creation of an AVF before dialysis initiation.** Two hundred twenty-one patients who received an preemptive AVF were included, of which 114 patients in belong to the AVF created early group, 88 patients to the AVF created on time group, and 19 patients to the AVF created late group. Patients are censored if they are lost to follow-up without reaching an event. Events are death and starting hemodialysis, whichever comes first.

## Discussion

This study reports on the timing of vascular access creation in relation to the start of hemodialysis treatment in six European countries. Only 32% of elderly ESKD patients started hemodialysis using an AVF that was created within the 6-month window. Twenty-eight percent of patients who received an AVF started hemodialysis with a CVC, which is associated with a higher risk of bloodstream infections.^5^ Conversely, 52% of the predialysis patients undergoing AVF creation did not initiate hemodialysis for at least 6 months after surgery, subjecting these patients to a longer time at risk for vascular access associated complications. The high rate of early AVF creation may, in part, be explained by the slower decline in eGFR among elderly patients.^11^

Vascular access surgery timing has previously been investigated in two studies from North America. A Canadian study examined AVF creation and hemodialysis initiation. The mean age (66 years) was lower than that of the VAST cohort, while the prevalence of diabetes mellitus was higher (55% versus 45%). However, the results are comparable with those of our cohort, with a median time to dialysis initiation of 6.1 months. In the Canadian study, 81% of patients initiated hemodialysis within the 2-year follow-up period after AVF creation.^6^ By contrast, the proportion of patients initiating hemodialysis after AVF creation was lower in the United States. Medicare cohort which included patients aged 70 years or older. In this Medicare cohort, 67% of patients started hemodialysis within 2 years, compared with 79% in our cohort. In addition, 15% of patients in the Medicare cohort died before initiating hemodialysis versus 7% in our study. Although the mean age was similar (78 years), the Medicare cohort included fewer male participants (54% versus 77%). Notably, comorbidities were more prevalent in the Medicare cohort, including diabetes (66% versus 46%), congestive heart failure (41% versus 17%), and peripheral vascular disease (63% versus 16%).^7^ These differences in comorbidity and mortality may explain the lower proportion of patients initiating hemodialysis in the Medicare cohort.

Although the vast majority of elderly patients eventually use their AVF, many are exposed to the potential complications of CVCs or unused AVFs because the vascular access is created outside the recommended time window. Our findings suggest that patients with less favorable biochemical characteristics, that is, lower hemoglobin and albumin or higher phosphate concentrations, tend to undergo AVF creation later. This may not only reflect planning but also the illness severity of these patients, who are often approaching KRT. Indeed, these biochemical parameters associated with the timing of AVF creation were similar to those included in the kidney failure risk equation (KFRE) that predicts the risk of kidney failure within 2- and 5-year periods.^12^ The actual timing of hemodialysis initiation is currently predominantly guided by factors such as eGFR, uremic symptoms, and hypervolemia. To facilitate timely vascular access creation, the KFRE prediction model has been explored as a complement to eGFR levels. Retrospective studies suggest that incorporating a 2-year KFRE risk threshold of ≥40% alongside eGFR-based criteria could increase the proportion of patients initiating hemodialysis with a functional AVF while reducing premature vascular access creation.^13,14^

Owing to the competing risks of mortality or opting for conservative treatment, early AVF creation may have greater consequences in elderly patients than in younger individuals. Nevertheless, this study demonstrates that 90% of elderly patients who undergo AVF creation commence hemodialysis treatment and only 7% of patients die with an unused fistula within 3 years. On the other hand, 26% of the patients started hemodialysis treatment using a CVC. The use of a CVC is associated with a higher risk of mortality and bloodstream infections compared with AVFs and AVGs.^5^ However, a causal relationship is uncertain, as patient selection may account for up to two-thirds of the observed survival advantage among patients with a fistula.^15^ Notably, the risk of CVC-related complications appears similar or even lower in the elderly compared with younger patients.^16–18^ It has been argued that the higher chance of nonmaturation and the reduced long-term benefits of AVFs due to the limited life expectancy make elderly patients with ESKD ideal candidates for AVGs. This strategy is used infrequently in Europe and only 1.5% of patients received an AVG as initial vascular access in the EQUAL study. Further investigation is warranted to evaluate whether AVFs should be the primary vascular access choice for elderly patients. Several randomized controlled trials are currently being conducted on this topic.^19–23^

A limitation of this study is the selected nature of the study population, as inclusion in the EQUAL cohort indicates that these patients had access to predialysis nephrology care, which is associated with improved outcomes and a reduced incidence of acute hemodialysis initiation. However, when determining the optimal timing for predialysis vascular access surgery in elderly patients, the so-called crash landers cannot be taken into account. The original EQUAL cohort was also restricted to patients in whom vascular access creation was pursued, and it was further restricted by excluding 183 patients due to missing data or insufficient follow-up. This may have introduced selection and survivorship bias, as patients with greater frailty, limited life expectancy, or unfavorable vascular anatomy were less likely to be referred, thereby limiting the generalizability of the findings to patients considered suitable for AVF creation. A second limitation is the synthetic nature of the subgroup definitions, which were based on the timing of vascular access creation relative to hemodialysis initiation rather than distinct clinical phenotypes. Consequently, outcomes reflect not only physician decision making regarding AVF referral but also patient-specific factors, particularly vascular fitness for AVF maturation and unexpected progression of CKD. A third limitation is the absence of data on AVF patency, interventions for maturation, vascular access related complications, and ultrasound-based measures of AVF usability, precluding a comprehensive assessment of access function.

This study represents the largest prospective cohort with elderly patients across European countries reporting the timing of hemodialysis vascular access creation. A key strength of this study is the 3-year follow-up period, compared with the 1 or 2 years used in previous studies. The extended follow-up allows for the vast majority of patient events to be captured (only five patients were alive without dialysis after 3 years). The multinational design and broad inclusion criteria of the EQUAL cohort limit bias and enhance generalizability within the European context. Importantly, despite differences in health care systems, clinical practices, and patient demographics, we found no statistically significant differences in outcomes between the participating countries.

In conclusion, stratifying elderly patients with ESKD in Europe by timing of vascular access creation, we found that only 32% received an AVF on time (within 6 months before hemodialysis initiation). Early AVF creation occurred in 42% of patients, potentially contributing to the number of unused AVFs, whereas late AVF creation occurred in 26%, exposing patients to potential CVC-related risks. These findings highlight the challenge of timely AVF placement. Despite this challenge, clinicians seem very accurate in predicting which patients will eventually require hemodialysis treatment, as 90% of patients eventually started hemodialysis after AVF creation within the follow-up period. These findings may aid in discussing vascular access options with elderly patients and indicate the need to develop a clinical prediction tool for imminent hemodialysis initiation to guide the timing of vascular access surgery.

## Supplementary Material

## Data Availability

Data belong to a third party, and authors are not authorized to share the data. Identity of Third Party: EQUAL publication committee (contact: n.c.chesnaye@amsterdamumc.nl). Reason for Restriction: The data underlying this article are sensitive health data and cannot be shared publicly due to the General Data Protection Regulation. The data will be shared on reasonable request to the EQUAL publication committee.

## References

[1] Boerstra BA Boenink R Astley ME, . The ERA Registry Annual Report 2021: A Summary. Clin Kidney J. 2024;17(2):sfad281. doi:10.1093/ckj/sfad281 PMC11024806 38638342

[2] Schmidli J Widmer MK Basile C, . Editor's choice - vascular access: 2018 Clinical practice guidelines of the European society for vascular surgery (ESVS). Eur J Vasc Endovasc Surg. 2018;55(6):757–818. doi:10.1016/j.ejvs.2018.02.001 29730128

[3] Lok CE Huber TS Lee T, . KDOQI clinical practice guideline for vascular access: 2019 update. Am J Kidney Dis. 2020;75(4 suppl 2):S1–S164. doi:10.1053/j.ajkd.2019.12.001 32778223

[4] Bylsma LC Gage SM Reichert H Dahl SLM Lawson JH. Arteriovenous fistulae for haemodialysis: a systematic review and meta-analysis of efficacy and safety outcomes. Eur J Vasc Endovasc Surg. 2017;54(4):513–522. doi:10.1016/j.ejvs.2017.06.024 28843984

[5] Ravani P Palmer SC Oliver MJ, . Associations between hemodialysis access type and clinical outcomes: a systematic review. J Am Soc Nephrol. 2013;24(3):465–473. doi:10.1681/ASN.2012070643 PMC3582202 23431075

[6] Oliver MJ Quinn RR Garg AX Kim SJ Wald R Paterson JM. Likelihood of starting dialysis after incident fistula creation. Clin J Am Soc Nephrol. 2012;7(3):466–471. doi:10.2215/CJN.08920811 PMC3302676 22344512

[7] Lee T Thamer M Zhang Y Zhang Q Allon M. Outcomes of elderly patients after predialysis vascular access creation. J Am Soc Nephrol. 2015;26(12):3133–3140. doi:10.1681/asn.2014090938 PMC4657836 25855782

[8] Jager KJ Ocak G Drechsler C, . The EQUAL study: a European study in chronic kidney disease stage 4 patients. Nephrol Dial Transplant. 2012;27(suppl 3):iii27–iii31. doi:10.1093/ndt/gfs277 22764196

[9] Janmaat CJ van Diepen M Meuleman Y, . Kidney function and symptom development over time in elderly patients with advanced chronic kidney disease: results of the EQUAL cohort study. Nephrol Dial Transplant. 2021;36(5):862–870. doi:10.1093/ndt/gfz277 PMC8075370 31943084

[10] Verduijn M Grootendorst DC Dekker FW Jager KJ le Cessie S. The analysis of competing events like cause-specific mortality--beware of the Kaplan-Meier method. Nephrol Dial Transplant. 2011;26(1):56–61. doi:10.1093/ndt/gfq661 21059831

[11] O'Hare AM Choi AI Bertenthal D, . Age affects outcomes in chronic kidney disease. J Am Soc Nephrol. 2007;18(10):2758–2765. doi:10.1681/ASN.2007040422 17855638

[12] Tangri N Stevens LA Griffith J, . A predictive model for progression of chronic kidney disease to kidney failure. JAMA. 2011;305(15):1553–1559. doi:10.1001/jama.2011.451 21482743

[13] Ingwiller M Keller N Krummel T, . Enhancing vascular access planning in CKD: validating the 40% KFRE threshold for predicting ESKD in a French retrospective cohort study. Clin Kidney J. 2024;17(8):sfae220. doi:10.1093/ckj/sfae220 PMC11483627 39421238

[14] Atiquzzaman M Zhu B Romann A, . Kidney failure risk equation in vascular access planning: a population-based study supporting value in decision making. Clin Kidney J. 2024;17(2):sfae008. doi:10.1093/ckj/sfae008 PMC10847629 38327282

[15] Brown RS Patibandla BK Goldfarb-Rumyantzev AS. The survival benefit of “fistula first, catheter last” in hemodialysis is primarily due to patient factors. J Am Soc Nephrol. 2017;28(2):645–652. doi:10.1681/ASN.2016010019 PMC5280011 27605542

[16] Poinen K Quinn RR Clarke A, . Complications from tunneled hemodialysis catheters: a Canadian Observational Cohort Study. Am J Kidney Dis. 2019;73(4):467–475. doi:10.1053/j.ajkd.2018.10.014 30642607

[17] Murea M James KM Russell GB, . Risk of catheter-related bloodstream infection in elderly patients on hemodialysis. Clin J Am Soc Nephrol. 2014;9(4):764–770. doi:10.2215/CJN.07710713 PMC3974362 24651074

[18] van Oevelen M Heggen BD Abrahams AC, . Central venous catheter-related complications in older haemodialysis patients: a multicentre observational cohort study. J Vasc Access. 2023;24(6):1322–1331. doi:10.1177/11297298221085225 PMC10714686 35360988

[19] Sengoelge G. Austrian Randomized Interventional Study on Dialysis Accesses (AUSTRIA). ClinicalTrials.gov; 2018. Accessed September, 2024.

[20] Peden E. Hemodialysis in the Elderly (70Yrs & Older) ClinicalTrials.gov; 2023. Accessed September, 2024.

[21] Murea M Geary RL Houston DK, . A randomized pilot study to evaluate graft versus fistula vascular access strategy in older patients with advanced kidney disease: results of a feasibility study. Pilot Feasibility Stud. 2020;6:86. doi:10.1186/s40814-020-00619-9 PMC7298797 32551134

[22] Quinn R Ravani P. ACCESS HD Investigators. ACCESS HD pilot: a randomised feasibility trial comparing catheters with fistulas in elderly patients starting haemodialysis. BMJ Open. 2016;6(11):e013081. doi:10.1136/bmjopen-2016-013081 PMC5168520 27884849

[23] Heggen BD Ramspek CL van der Bogt KEA, . Optimising access surgery in senior haemodialysis patients (OASIS): study protocol for a multicentre randomised controlled trial. BMJ Open. 2022;12(2):e053108. doi:10.1136/bmjopen-2021-053108 PMC8814743 35115352

