# Supplementary material for: Vascular Access Surgery Timing: A Prospective European Cohort Study in Elderly Patients with ESKD
Source: Kidney360. 2026 Mar 27;7(9):2066–74. doi: 10.34067/KID.0000001180 (PMC13641530; doi:10.34067/KID.0000001180)
Supplement: Supplementary file 1 [file kidney360-7-2066-s001.pdf]

ASN
American Society of Nephrology
JASN
Journal of the American Society of Nephrology
KIDNEY360
Accessing Our World From Every Angle
CJASN
Clinical Journal of the American Society of Nephrology

ASN Journal Disclosure Form

As per ASN journal policy, I have disclosed any financial relationships or commitments I have held in the past 36 months as included below. I have listed my Current Employer below to indicate there is a relationship requiring disclosure. If no relationship exists, my Current Employer is not listed.

F. Caskey reports the following:
Employer: University of Bristol; Research Funding: National Institute for Health Research; Kidney Research UK; PCORI, LifeArc.; and Advisory or Leadership Role: International Society of Nephrology (Treasurer, Honorary Secretary, Executive Committee member). Unpaid.

I understand that the information above will be published within the journal article, if accepted, and that failure to comply and/or to accurately and completely report the potential financial conflicts of interest could lead to the following: 1) Prior to publication, article rejection, or 2) Post-publication, sanctions ranging from, but not limited to, issuing a correction, reporting the inaccurate information to the authors' institution, banning authors from submitting work to ASN journals for varying lengths of time, and/or retraction of the published work.

Name: Fergus Caskey
Manuscript ID: K360-2025-001315R2
Manuscript Title: Vascular Access Surgery Timing: A prospective European cohort study in elderly patients with end-stage kidney disease
Date of Completion: February 6, 2026
Disclosure Updated Date: January 22, 2026

ASN American Society of Nephrology JASN Journal of the American Society of Nephrology KIDNEY360 Accessing Our World From Every Angle CJASN Clinical Journal of the American Society of Nephrology

ASN Journal Disclosure Form

As per ASN journal policy, I have disclosed any financial relationships or commitments I have held in the past 36 months as included below. I have listed my Current Employer below to indicate there is a relationship requiring disclosure. If no relationship exists, my Current Employer is not listed.

N. Chesnaye reports the following:
Employer: Amsterdam UMC

I understand that the information above will be published within the journal article, if accepted, and that failure to comply and/or to accurately and completely report the potential financial conflicts of interest could lead to the following: 1) Prior to publication, article rejection, or 2) Post-publication, sanctions ranging from, but not limited to, issuing a correction, reporting the inaccurate information to the authors' institution, banning authors from submitting work to ASN journals for varying lengths of time, and/or retraction of the published work.

Name: Nicholas C. Chesnaye
Manuscript ID: K360-2025-001315R1
Manuscript Title: Vascular Access Surgery Timing: A prospective European cohort study in elderly patients with end-stage kidney disease
Date of Completion: January 22, 2026
Disclosure Updated Date: January 22, 2026

ASN American Society of Nephrology | JASN Journal of the American Society of Nephrology | KIDNEY360 Accessing Our World From Every Angle | CJASN Clinical Journal of the American Society of Nephrology

ASN Journal Disclosure Form

As per ASN journal policy, I have disclosed any financial relationships or commitments I have held in the past 36 months as included below. I have listed my Current Employer below to indicate there is a relationship requiring disclosure. If no relationship exists, my Current Employer is not listed.

F. Dekker reports the following:
Research Funding: CSL-Vifor

I understand that the information above will be published within the journal article, if accepted, and that failure to comply and/or to accurately and completely report the potential financial conflicts of interest could lead to the following: 1) Prior to publication, article rejection, or 2) Post-publication, sanctions ranging from, but not limited to, issuing a correction, reporting the inaccurate information to the authors' institution, banning authors from submitting work to ASN journals for varying lengths of time, and/or retraction of the published work.

Name: Friedo W. Dekker
Manuscript ID: K360-2025-001315R2
Manuscript Title: Vascular Access Surgery Timing: A prospective European cohort study in elderly patients with end-stage kidney disease
Date of Completion: February 13, 2026
Disclosure Updated Date: February 13, 2026

ASN American Society of Nephrology
JASN Journal of the American Society of Nephrology
KIDNEY360 Accessing Our World From Every Angle
CJASN Clinical Journal of the American Society of Nephrology

ASN Journal Disclosure Form

As per ASN journal policy, I have disclosed any financial relationships or commitments I have held in the past 36 months as included below. I have listed my Current Employer below to indicate there is a relationship requiring disclosure. If no relationship exists, my Current Employer is not listed.

C. Drechsler reports the following:
Honoraria: Boehringer Ingelheim; and Advisory or Leadership Role: Boehringer Ingelheim.

I understand that the information above will be published within the journal article, if accepted, and that failure to comply and/or to accurately and completely report the potential financial conflicts of interest could lead to the following: 1) Prior to publication, article rejection, or 2) Post-publication, sanctions ranging from, but not limited to, issuing a correction, reporting the inaccurate information to the authors' institution, banning authors from submitting work to ASN journals for varying lengths of time, and/or retraction of the published work.

Name: Christiane Drechsler
Manuscript ID: K360-2025-001315R1
Manuscript Title: Vascular Access Surgery Timing: A prospective European cohort study in elderly patients with end-stage kidney disease
Date of Completion: January 22, 2026
Disclosure Updated Date: January 22, 2026

ASN
American Society of Nephrology
JASN
Journal of the American Society of Nephrology
KIDNEY360
Accessing Our World From Every Angle
CJASN
Clinical Journal of the American Society of Nephrology

ASN Journal Disclosure Form

As per ASN journal policy, I have disclosed any financial relationships or commitments I have held in the past 36 months as included below. I have listed my Current Employer below to indicate there is a relationship requiring disclosure. If no relationship exists, my Current Employer is not listed.

M. Evans reports the following:
Employer: Karolinska Institutet and; Karolinska University hospital; Research Funding: Astellas pharma and Astra Zeneca, institutional grant; Honoraria: Payment for lectures by Astellas, Astra Zeneca, Vifor Pharma, and Boehringer-Ingelheim; and Other Interests or Relationships: Member of Steering committee in the Swedish Renal Registry.

I understand that the information above will be published within the journal article, if accepted, and that failure to comply and/or to accurately and completely report the potential financial conflicts of interest could lead to the following: 1) Prior to publication, article rejection, or 2) Post-publication, sanctions ranging from, but not limited to, issuing a correction, reporting the inaccurate information to the authors' institution, banning authors from submitting work to ASN journals for varying lengths of time, and/or retraction of the published work.

Name: Marie Evans
Manuscript ID: K360-2025-001315R3
Manuscript Title: Vascular Access Surgery Timing: A prospective European cohort study in elderly patients with end-stage kidney disease.
Date of Completion: March 5, 2026
Disclosure Updated Date: August 26, 2025

ASN
American Society of Nephrology
JASN
Journal of the American Society of Nephrology
KIDNEY360
Accessing Our World From Every Angle
CJASN
Clinical Journal of the American Society of Nephrology

ASN Journal Disclosure Form

As per ASN journal policy, I have disclosed any financial relationships or commitments I have held in the past 36 months as included below. I have listed my Current Employer below to indicate there is a relationship requiring disclosure. If no relationship exists, my Current Employer is not listed.

B. Heggen reports the following:
Employer: Maastricht University Medical Center+; Maxima Medical Center; Ownership Interest: Heggen Beheer BV; and Research Funding: Leading the Change (ZonMw project number 80-85009-98-2004).

I understand that the information above will be published within the journal article, if accepted, and that failure to comply and/or to accurately and completely report the potential financial conflicts of interest could lead to the following: 1) Prior to publication, article rejection, or 2) Post-publication, sanctions ranging from, but not limited to, issuing a correction, reporting the inaccurate information to the authors' institution, banning authors from submitting work to ASN journals for varying lengths of time, and/or retraction of the published work.

Name: Boudewijn Heggen
Manuscript ID: K360-2025-001315R2
Manuscript Title: Vascular Access Surgery Timing: A prospective European cohort study in elderly patients with end-stage kidney disease.
Date of Completion: February 12, 2026
Disclosure Updated Date: February 12, 2026

ASN American Society of Nephrology | JASN Journal of the American Society of Nephrology | KIDNEY360 Accessing Our World From Every Angle | CJASN Clinical Journal of the American Society of Nephrology

ASN Journal Disclosure Form

As per ASN journal policy, I have disclosed any financial relationships or commitments I have held in the past 36 months as included below. I have listed my Current Employer below to indicate there is a relationship requiring disclosure. If no relationship exists, my Current Employer is not listed.

M. Hiligsmann reports the following:
Employer: Maastricht University; Consultancy: Grant advisor (paid to institution from Pfizer and research advisor (paid to institution) from Grunenthal and Teva.; Research Funding: Grant research from Angelini Pharma (paid to institution); Advisory or Leadership Role: Editor in chief of Expert Review of Pharmacoeconomics & Outcomes Research (paid to institution); and Speakers Bureau: lecture fee from Echolight.

I understand that the information above will be published within the journal article, if accepted, and that failure to comply and/or to accurately and completely report the potential financial conflicts of interest could lead to the following: 1) Prior to publication, article rejection, or 2) Post-publication, sanctions ranging from, but not limited to, issuing a correction, reporting the inaccurate information to the authors' institution, banning authors from submitting work to ASN journals for varying lengths of time, and/or retraction of the published work.

Name: Mickaël Hiligsmann
Manuscript ID: K360-2025-001315R1
Manuscript Title: Vascular Access Surgery Timing: A prospective European cohort study in elderly patients with end-stage kidney disease
Date of Completion: January 22, 2026
Disclosure Updated Date: January 22, 2026

ASN Journal Disclosure Form

As per ASN journal policy, I have disclosed any financial relationships or commitments I have held in the past 36 months as included below. I have listed my Current Employer below to indicate there is a relationship requiring disclosure. If no relationship exists, my Current Employer is not listed.

K. Jager reports the following:
Employer: Amsterdam University Medical Center; and Advisory or Leadership Role: - Council Member European Renal Association; - Editorial Boards of Nephrology Dialysis Transplantation and African Journal of Nephrology; all unpaid.

I understand that the information above will be published within the journal article, if accepted, and that failure to comply and/or to accurately and completely report the potential financial conflicts of interest could lead to the following: 1) Prior to publication, article rejection, or 2) Post-publication, sanctions ranging from, but not limited to, issuing a correction, reporting the inaccurate information to the authors' institution, banning authors from submitting work to ASN journals for varying lengths of time, and/or retraction of the published work.

Name: Kitty J. Jager
Manuscript ID: K360-2025-001315R1
Manuscript Title: Vascular Access Surgery Timing: A prospective European cohort study in elderly patients with end-stage kidney disease
Date of Completion: January 23, 2026
Disclosure Updated Date: December 23, 2025

ASN American Society of Nephrology | JASN Journal of the American Society of Nephrology | KIDNEY360 Accessing Our World From Every Angle | CJASN Clinical Journal of the American Society of Nephrology

ASN Journal Disclosure Form

As per ASN journal policy, I have disclosed any financial relationships or commitments I have held in the past 36 months as included below. I have listed my Current Employer below to indicate there is a relationship requiring disclosure. If no relationship exists, my Current Employer is not listed.

M. Pippias reports the following:
Employer: North Bristol NHS Trust; and Advisory or Leadership Role: ISN SharE-RR co-chair.

I understand that the information above will be published within the journal article, if accepted, and that failure to comply and/or to accurately and completely report the potential financial conflicts of interest could lead to the following: 1) Prior to publication, article rejection, or 2) Post-publication, sanctions ranging from, but not limited to, issuing a correction, reporting the inaccurate information to the authors' institution, banning authors from submitting work to ASN journals for varying lengths of time, and/or retraction of the published work.

Name: Maria Pippias
Manuscript ID: K360-2025-001315R2
Manuscript Title: Vascular Access Surgery Timing: A prospective European cohort study in elderly patients with end-stage kidney disease.
Date of Completion: February 8, 2026
Disclosure Updated Date: January 22, 2026

ASN
American Society of Nephrology
JASN
Journal of the American Society of Nephrology
KIDNEY360
Accessing Our World From Every Angle
CJASN
Clinical Journal of the American Society of Nephrology

ASN Journal Disclosure Form

As per ASN journal policy, I have disclosed any financial relationships or commitments I have held in the past 36 months as included below. I have listed my Current Employer below to indicate there is a relationship requiring disclosure. If no relationship exists, my Current Employer is not listed.

J. Rotmans reports the following:
Employer: LUMC; Consultancy: Xeltis BV; Ownership Interest: XS Innovations; Research Funding: AstraZeneca; Advisory or Leadership Role: past-president Vascular Access Society; Advisory Board Nextkidney; and Other Interests or Relationships: Chair Thematic Working Group Vascular Tissue Engineering at TERMIS.

I understand that the information above will be published within the journal article, if accepted, and that failure to comply and/or to accurately and completely report the potential financial conflicts of interest could lead to the following: 1) Prior to publication, article rejection, or 2) Post-publication, sanctions ranging from, but not limited to, issuing a correction, reporting the inaccurate information to the authors' institution, banning authors from submitting work to ASN journals for varying lengths of time, and/or retraction of the published work.

Name: Joris I. Rotmans
Manuscript ID: K360-2025-001315R2
Manuscript Title: Vascular Access Surgery Timing: A prospective European cohort study in elderly patients with end-stage kidney disease
Date of Completion: February 10, 2026
Disclosure Updated Date: January 30, 2026

ASN American Society of Nephrology JASN Journal of the American Society of Nephrology KIDNEY360 Accessing Our World From Every Angle CJASN Clinical Journal of the American Society of Nephrology

ASN Journal Disclosure Form

As per ASN journal policy, I have disclosed any financial relationships or commitments I have held in the past 36 months as included below. I have listed my Current Employer below to indicate there is a relationship requiring disclosure. If no relationship exists, my Current Employer is not listed.

G. Schurink reports the following:
Employer: MUMC

I understand that the information above will be published within the journal article, if accepted, and that failure to comply and/or to accurately and completely report the potential financial conflicts of interest could lead to the following: 1) Prior to publication, article rejection, or 2) Post-publication, sanctions ranging from, but not limited to, issuing a correction, reporting the inaccurate information to the authors' institution, banning authors from submitting work to ASN journals for varying lengths of time, and/or retraction of the published work.

Name: Geert willem H. Schurink
Manuscript ID: K360-2025-001315R3
Manuscript Title: Vascular Access Surgery Timing: A prospective European cohort study in elderly patients with end-stage kidney disease
Date of Completion: February 26, 2026
Disclosure Updated Date: January 22, 2026

ASN
American Society of Nephrology
JASN
Journal of the American Society of Nephrology
KIDNEY360
Accessing Our World From Every Angle
CJASN
Clinical Journal of the American Society of Nephrology

ASN Journal Disclosure Form

As per ASN journal policy, I have disclosed any financial relationships or commitments I have held in the past 36 months as included below. I have listed my Current Employer below to indicate there is a relationship requiring disclosure. If no relationship exists, my Current Employer is not listed.

M. Snoeijs reports the following:
Employer: Maastricht University Medical Center; and Research Funding: Merit Medical (compensation for participation in industry-initiated registry); Xeltis (compensation for participation in industry-initiated first-in-man trial).

I understand that the information above will be published within the journal article, if accepted, and that failure to comply and/or to accurately and completely report the potential financial conflicts of interest could lead to the following: 1) Prior to publication, article rejection, or 2) Post-publication, sanctions ranging from, but not limited to, issuing a correction, reporting the inaccurate information to the authors' institution, banning authors from submitting work to ASN journals for varying lengths of time, and/or retraction of the published work.

Name: Maarten G. Snoeijs
Manuscript ID: K360-2025-001315R2
Manuscript Title: Vascular Access Surgery Timing: A prospective European cohort study in elderly patients with end-stage kidney disease
Date of Completion: February 6, 2026
Disclosure Updated Date: January 21, 2026

ASN
American Society of Nephrology
JASN
Journal of the American Society of Nephrology
KIDNEY360
Accessing Our World From Every Angle
CJASN
Clinical Journal of the American Society of Nephrology

ASN Journal Disclosure Form

As per ASN journal policy, I have disclosed any financial relationships or commitments I have held in the past 36 months as included below. I have listed my Current Employer below to indicate there is a relationship requiring disclosure. If no relationship exists, my Current Employer is not listed.

M. Szymczak reports the following:
Employer: Wroclaw Medical University

I understand that the information above will be published within the journal article, if accepted, and that failure to comply and/or to accurately and completely report the potential financial conflicts of interest could lead to the following: 1) Prior to publication, article rejection, or 2) Post-publication, sanctions ranging from, but not limited to, issuing a correction, reporting the inaccurate information to the authors' institution, banning authors from submitting work to ASN journals for varying lengths of time, and/or retraction of the published work.

Name: Maciej Szymczak
Manuscript ID: K360-2025-001315R1
Manuscript Title: Vascular Access Surgery Timing: A prospective European cohort study in elderly patients with end-stage kidney disease.
Date of Completion: January 21, 2026
Disclosure Updated Date: January 21, 2026

ASN Journal Disclosure Form

As per ASN journal policy, I have disclosed any financial relationships or commitments I have held in the past 36 months as included below. I have listed my Current Employer below to indicate there is a relationship requiring disclosure. If no relationship exists, my Current Employer is not listed.

C. Torino reports the following:
Employer: IFC-CNR Reggio Calabria

I understand that the information above will be published within the journal article, if accepted, and that failure to comply and/or to accurately and completely report the potential financial conflicts of interest could lead to the following: 1) Prior to publication, article rejection, or 2) Post-publication, sanctions ranging from, but not limited to, issuing a correction, reporting the inaccurate information to the authors' institution, banning authors from submitting work to ASN journals for varying lengths of time, and/or retraction of the published work.

Name: Claudia Torino
Manuscript ID: K360-2025-001315R3
Manuscript Title: Vascular Access Surgery Timing: A prospective European cohort study in elderly patients with end-stage kidney disease
Date of Completion: February 26, 2026
Disclosure Updated Date: January 22, 2026

ASN Journal Disclosure Form

As per ASN journal policy, I have disclosed any financial relationships or commitments I have held in the past 36 months as included below. I have listed my Current Employer below to indicate there is a relationship requiring disclosure. If no relationship exists, my Current Employer is not listed.

M. van Diepen reports the following:
Employer: Leiden University Medical Center; and Other Interests or Relationships: This research was supported by a grant from the Dutch Kidney Foundation (20OK016).

I understand that the information above will be published within the journal article, if accepted, and that failure to comply and/or to accurately and completely report the potential financial conflicts of interest could lead to the following: 1) Prior to publication, article rejection, or 2) Post-publication, sanctions ranging from, but not limited to, issuing a correction, reporting the inaccurate information to the authors' institution, banning authors from submitting work to ASN journals for varying lengths of time, and/or retraction of the published work.

Name: Merel van Diepen
Manuscript ID: K360-2025-001315R1
Manuscript Title: Vascular Access Surgery Timing: A prospective European cohort study in elderly patients with end-stage kidney disease
Date of Completion: January 26, 2026
Disclosure Updated Date: January 26, 2026

ASN American Society of Nephrology JASN Journal of the American Society of Nephrology KIDNEY360 Accessing Our World From Every Angle CJASN Clinical Journal of the American Society of Nephrology

ASN Journal Disclosure Form

As per ASN journal policy, I have disclosed any financial relationships or commitments I have held in the past 36 months as included below. I have listed my Current Employer below to indicate there is a relationship requiring disclosure. If no relationship exists, my Current Employer is not listed.

A. Vilasi reports the following:
Employer: IFC-CNR, sede secondaria di Reggio Calabria

I understand that the information above will be published within the journal article, if accepted, and that failure to comply and/or to accurately and completely report the potential financial conflicts of interest could lead to the following: 1) Prior to publication, article rejection, or 2) Post-publication, sanctions ranging from, but not limited to, issuing a correction, reporting the inaccurate information to the authors' institution, banning authors from submitting work to ASN journals for varying lengths of time, and/or retraction of the published work.

Name: Antonio Vilasi
Manuscript ID: K360-2025-001315R2
Manuscript Title: Vascular Access Surgery Timing: A prospective European cohort study in elderly patients with end-stage kidney disease
Date of Completion: February 11, 2026
Disclosure Updated Date: February 11, 2026

ASN American Society of Nephrology | JASN Journal of the American Society of Nephrology | KIDNEY360 Accessing Our World From Every Angle | CJASN Clinical Journal of the American Society of Nephrology

ASN Journal Disclosure Form

As per ASN journal policy, I have disclosed any financial relationships or commitments I have held in the past 36 months as included below. I have listed my Current Employer below to indicate there is a relationship requiring disclosure. If no relationship exists, my Current Employer is not listed.

C. Wanner reports the following:
Employer: University Hospital; Consultancy: Alexion, AstraZeneca, Bayer, Boehringer-Ingelheim, GSK, Idorsia, MSD, NovoNordisk, CSL-Vifor, VeraTX; Research Funding: University of Oxford, Sanofi, AstraZeneca; and Honoraria: Amgen, Amicus, Astellas, AstraZeneca, Bayer, Boehringer-Ingelheim, Chiesi, FMC, Eli-Lilly, GSK, Novartis, Pfizer, Sanofi, Stadapharm, Takeda, CSL-Vifor.

I understand that the information above will be published within the journal article, if accepted, and that failure to comply and/or to accurately and completely report the potential financial conflicts of interest could lead to the following: 1) Prior to publication, article rejection, or 2) Post-publication, sanctions ranging from, but not limited to, issuing a correction, reporting the inaccurate information to the authors' institution, banning authors from submitting work to ASN journals for varying lengths of time, and/or retraction of the published work.

Name: Christoph Wanner
Manuscript ID: K360-2025-001315R2
Manuscript Title: Vascular Access Surgery Timing: A prospective European cohort study in elderly patients with end-stage kidney disease
Date of Completion: February 7, 2026
Disclosure Updated Date: January 21, 2026
